# Widespread zoophagy and detection of *Plasmodium* spp. in *Anopheles* mosquitoes in southeastern Madagascar

**DOI:** 10.1186/s12936-020-03539-4

**Published:** 2021-01-07

**Authors:** Micaela Finney, Benjamin A. McKenzie, Bernadette Rabaovola, Alice Sutcliffe, Ellen Dotson, Sarah Zohdy

**Affiliations:** 1 College of Science and Mathematics, Auburn University, Auburn, AL USA; 2 School of Forestry and Wildlife Sciences, Auburn University, Auburn, AL USA; 3 Centre Val Bio, Ranomafana, Madagascar; 4 Division of Parasitic Diseases and Malaria, Entomology Branch, Centers for Disease Control and Prevention, Atlanta, GA USA; 5 College of Veterinary Medicine, Auburn University, Auburn, AL USA; 6 Present Address: Entomology Department, College of Agriculture, Texas A&M University, College Station, TX USA

**Keywords:** Blood meal, Cattle, Livestock, *Plasmodium vivax*, Mosquito, Ranomafana

## Abstract

**Background:**

Malaria is a top cause of mortality on the island nation of Madagascar, where many rural communities rely on subsistence agriculture and livestock production. Understanding feeding behaviours of *Anopheles* in this landscape is crucial for optimizing malaria control and prevention strategies. Previous studies in southeastern Madagascar have shown that *Anopheles* mosquitoes are more frequently captured within 50 m of livestock. However, it remains unknown whether these mosquitoes preferentially feed on livestock. Here, mosquito blood meal sources and *Plasmodium* sporozoite rates were determined to evaluate patterns of feeding behaviour in *Anopheles* spp. and malaria transmission in southeastern Madagascar.

**Methods:**

Across a habitat gradient in southeastern Madagascar 7762 female *Anopheles* spp. mosquitoes were collected. Of the captured mosquitoes, 492 were visibly blood fed and morphologically identifiable, and a direct enzyme-linked immunosorbent assay (ELISA) was used to test for swine, cattle, chicken, human, and dog blood among these specimens. Host species identification was confirmed for multiple blood meals using PCR along with Sanger sequencing. Additionally, 1,607 *Anopheles* spp. were screened for the presence of *Plasmodium falciparum*, *P. vivax*-210, and *P. vivax* 247 circumsporozoites (cs) by ELISA.

**Results:**

Cattle and swine accounted, respectively, for 51% and 41% of all blood meals, with the remaining 8% split between domesticated animals and humans. Of the 1,607 *Anopheles* spp. screened for *Plasmodium falciparum, Plasmodium vivax* 210*,* and *Plasmodium vivax* 247 cs-protein, 45 tested positive, the most prevalent being *P. vivax* 247, followed by *P. vivax* 210 and *P. falciparum*. Both variants of *P. vivax* were observed in secondary vectors, including *Anopheles squamosus/cydippis*, *Anopheles coustani*, and unknown *Anopheles* spp. Furthermore, evidence of coinfection of *P. falciparum* and *P. vivax* 210 in *Anopheles gambiae *sensu lato (*s.l.*) was found.

**Conclusions:**

Here, feeding behaviour of *Anopheles* spp. mosquitoes in southeastern Madagascar was evaluated, in a livestock rich landscape. These findings suggest largely zoophagic feeding behaviors of *Anopheles* spp., including *An. gambiae s.l.* and presence of both *P. vivax and P. falciparum* sporozoites in *Anopheles* spp. A discordance between *P. vivax* reports in mosquitoes and humans exists, suggesting high prevalence of *P. vivax* circulating in vectors in the ecosystem despite low reports of clinical vivax malaria in humans in Madagascar. Vector surveillance of *P. vivax* may be relevant to malaria control and elimination efforts in Madagascar. At present, the high proportion of livestock blood meals in Madagascar may play a role in buffering (zooprophylaxis) or amplifying (zoopotentiation) the impacts of malaria. With malaria vector control efforts focused on indoor feeding behaviours, complementary approaches, such as endectocide-aided vector control in livestock may be an effective strategy for malaria reduction in Madagascar.

## Background

Human malaria, caused by an infection with *Plasmodium* parasites and transmitted by *Anopheles* mosquitoes, is one of the greatest causes of mortality in the world. Nearly half-a-million people die from malaria each year, with the greatest burden (> 90%) of morbidity and mortality occurring in sub-Saharan Africa [1]. Malaria control efforts have increased in recent years, with a resurgence in investment in research and practical prevention efforts such as targeted vector control strategies. These targeted vector control strategies use insecticide-treated bed nets (ITNs), as well as indoor residual spraying (IRS) to reduce vector populations and to protect humans from malaria transmission via mosquito bites [2]. The overwhelming focus on insecticides, in particular with ITNs and IRS, for malaria prevention over the past several decades has led to shifts in mosquito susceptibility to these insecticides [3–7], and may lead to facultative or long-term shifts in the behaviour of vector species [8–12]. Most populations of the primary malaria vector species in Africa, *Anopheles gambiae *sensu stricto (*s.s*.) and *Anopheles funestus s.s.*, are now at least partially resistant to most pyrethroid insecticides [11, 12], while development of resistance to other commonly used classes of insecticides, such as carbamates and organophosphates, is ongoing [11, 13]. Certain populations of *An. gambiae *sensu lato (*s.l*.) and *An. funestus s.l*. also seem to have undergone behavioural changes following implementation of ITN campaigns, switching to feeding at times when people are less likely to be under bed nets [4, 14, 15] as well as displaying increased levels of exophagy, avoiding contact with insecticide while seeking a host [4, 6, 7]. In regions where *Anopheles* vectors are becoming increasingly exophagic, they also appear to be becoming increasingly zoophagic [3, 6, 7, 16, 17], supporting research which indicates that extrinsic factors such as host availability influence host blood meal preference, although methodological variables, such as indoor/outdoor trapping location may influence the type of blood meal detected [18, 19]. While measures such as ITNs are effective in reducing the bulk of human malaria cases caused by endophagic mosquitoes, residual transmission, facilitated by exophagic mosquitoes, remains an issue of concern [6, 16, 20–23]. In order to achieve long-term malaria control and to fight residual transmission, it is critical to consider mosquito ecology and to integrate alternative vector control strategies with the methods currently used [20, 24, 25].

The island nation of Madagascar represents an area of ongoing residual malaria transmission where alternative strategies may need to be integrated with current approaches to combat transmission. One hundred percent of Madagascar’s population lives in areas where malaria is endemic [26]. Despite successful control strategies in Madagascar in recent decades, the impact of ongoing efforts to distribute ITNs, as well as widespread application of IRS, has not been enough to halt the transmission of malaria-causing parasites. This is evident in the extensive outbreaks seen in recent years [27]. The continued malaria burden and impact on mortality in Madagascar suggests current vector control strategies need to be revised and improved. The high ratio of cattle to humans could create potential for a zooprophylactic approach, one in which livestock are used to divert malaria vectors from human populations. The success of such an approach depends on the importance of various vector species and their host feeding preferences.

The goals of this study were to: (1) determine community compositions of host-seeking malaria vector species in southeastern Madagascar, (2) determine host preference of *Anopheles* spp. collected in this area, where habitat alteration is common and livestock are prevalent, and (3) determine the proportion of *Anopheles* spp. in this area harbouring infective sporozoite stages of *Plasmodium* parasites *Plasmodium falciparum*, *Plasmodium vivax* VK210 (*P. vivax* 210) and *P. vivax* VK247 (*P. vivax* 247). Due to the high level of zoophagy demonstrated by *Anopheles* in other parts of sub-Saharan Africa where livestock are common, it was hypothesized that in this study area, mosquito species which are typically considered to be anthropophagic, such as *An. gambiae s.l.* and *An. funestus s.l*., would exhibit feeding preference toward livestock, such as cattle. It was further hypothesized that typically zoophagic species, such as *Anopheles coustani* and *Anopheles squamosus/cydippis,* would maintain preference for feeding on livestock. Additionally, it was hypothesized that these zoophagic vectors would exhibit *Plasmodium* sporozoite rates similar to those of the primary vectors, *An. gambiae s.s.* and *An. funestus s.s.* Finally, based on reported case data, it was hypothesized that *P. vivax* would be present, but its prevalence in the vector population would be lower than that of *P. falciparum* [26].

## Methods

### Mosquito sampling and identification

Sampling was conducted in the Ifanadiana district of southeastern Madagascar (21° 02′–21° 25′ S, 47° 18′–47° 37′ E), a location characterized by a highly seasonal environment. Mosquito collections were done during both dry and rainy seasons, spanning August – December 2016 in seven villages; Namhoaka, Kianjanoby, Ambatavory, Ampasipotsy, Ambasoary, Mangevo, and Amboditanimena. Elevation varied from 116 to 533 m above sea level. Villages where traps were placed were selected based on the presence of livestock and close proximity to habitat altered through slash-and-burn agricultural techniques. All villages were within 1 km of the boundary of Ranomafana National Park (RNP), a 42500 hectare protected montane rainforest. Mosquitoes were collected using CDC miniature light traps baited with field-produced CO_2_ made from a sugar-yeast-water mixture. Specifically, the CO_2_ mixture was made by combining 1 part yeast with 3 parts sugar, gently mixed for 30 min, followed by the addition of 1 part brown sugar in a recycled 1.5 L bottle (adapted from [28]).

Twelve light traps were placed in each of the seven villages for three consecutive nights in each site, for a total of 252 “trap nights”. Traps were placed at 16:00 and checked at 07:00 the following day. In each village, four traps were set in sites within < 10 m of livestock, four inside houses, and four in forested sites near (< 1 km) the village centre or nearby rice fields. Mosquito collection bags were removed and replaced with new ones each afternoon. Captured mosquitoes were morphologically identified in the field to the genus level and *An. gambiae s.l*., *An. funestus s.l.,* and *An. coustani* using the key of Gillies and Coetzee [29]. Specimens were sorted into individual 1.5 mL tubes with desiccant and stored in a cool, dry place until they were brought to a laboratory and identified to species also using the Grjebine key [30]. Specimens within *An. gambiae* and *An. funestus* complexes were assayed using a protocol provided by Scott et al*.* [31] and Walker et al*.* [32]. All *Anopheles* spp. mosquitoes were dissected and stored separately as wings plus legs, abdomen, and head plus thorax. Mosquitoes that appeared to be blood fed using a stereomicroscope were identified for downstream blood meal analysis.

### Direct enzyme-linked immunosorbent assays (ELISAs) for blood meal detection

The abdomens of blood fed mosquitoes were ground in 50 µL of phosphate buffer saline (PBS) using a motor-powered pestle. After grinding, the pestle was rinsed with 450 µl of PBS and the eluate was collected into the original tube for a final volume of approximately 500 µl. Samples were stored overnight at 4 °C and tested the following day. Positive and negative controls were stored on desiccant at − 20 °C and were prepared in the same manner as described above for blood fed abdomens. Positive controls were generated by feeding previously unfed *An. gambiae* (BEI Resources MR4, MRA-112) using commercially purchased blood (Hemostat) on a membrane apparatus under laboratory conditions at the US Centers for Disease Control and Prevention. One positive control was used per assay. No positive control for dog was used. Seven unfed *An. gambiae s.l.* abdomens were used in each assay as negative controls.

The specifics of the direct ELISA and blocking buffer protocol can be found in the paper by Beier et al*.* [33]. The assay was run with host specific markers for: pig, cattle, dog, chicken, human (SeraCare product number: 5220–0363, 5220–0361, 5220–0368, 5220–0373, 522–0330, respectively). Plates were covered for all incubations to prevent evaporation and held at room temperature. All washes were performed using approximately 200 µL of PBS-T (0.5% Tween 20 in PBS). Briefly, 50 µl of each prepared sample were placed into a separate well of a 96-well assay plate (Costar) and incubated for three hours. The wells were then washed twice and then incubated with 50 µl of conjugate per well for one hour. The conjugate was incubated for three hours at 4 °C before use and consisted of host-specific peroxidase-labelled antibody and undiluted non-host serum to help control non-specific cross-reactivity. The total volume in the wells was aspirated out, washed three times and incubated with 100 µl ABTS (SeraCare 5120–0032, 50 µL component A and 50µL component B) for 30 min. Following incubation, absorbance was immediately measured at 405 nm with a SpectraMax 340 spectrophotometer (Molecular Devices, LLC., San Jose, CA) using Softmax Pro 7 software.

### DNA extraction from blood meal ELISA homogenate

Samples that included evidence of a blood meal from more than one host species using the direct ELISA were further analysed by multiplex PCR. DNA was extracted from the remaining mosquito abdomen ELISA homogenate according to Collins et al*.* [34]. This protocol typically starts with unprocessed mosquito tissue, thus a 2X concentrated extraction buffer was formulated resulting in a standard 1X concentration of all components when added to the abdomen homogenate. 200 µL of abdomen homogenate was combined with 200 µL of 2X Collins buffer and incubated at 65 °C for 90 min. Potassium acetate was added to a concentration of 1 M and samples were centrifuged at 2270*g* for 20 min at room temperature. The supernatant was transferred to a new tube mixed with 450 µL of ice cold 100% EtOH and incubated at 4 °C overnight. The next day, the tubes were centrifuged at 2270 g at 4 °C for 20 min, supernatant was discarded, and the remaining pellet was washed with 200µL of ice cold 70% EtOH. Following centrifugation at 2270*g* for 5 min and removal of supernatant, the tubes were placed in a speed vacuum concentrator for 20 min at room temperature to allow any remaining EtOH to evaporate. DNA was resuspended in 20µL of reagent-grade water and stored at 4 °C until use.

### Multiplex polymerase chain reaction (PCR)

The samples with more than one positive results from the blood meal ELISA were evaluated with a multiplex PCR targeting a region of cytochrome B DNA [35] using the following primers: PIG573F (PigF) (5′-CCT CGC AGC CGT ACA TCT C-3′), DOG368F (DogF) (5′-GGA ATT GTA CTA TTA TTC GCA ACC AT-3′), COW121F (CowF) (5′-CAT CGG CAC AAA TTT AGT CG-3′), HUMAN741F (HumF) (5′-GGC TTA CTT CTC TTC ATT CTC TCC T-3′), and UNREV1025 (5′-GGT TGT CCT CCA ATT CAT GTT A-3′). Total PCR reaction volumes were 25 µL and contained 0.1 µM of each primer, 2 × AccuStart™ II GelTrack PCR SuperMix and 1.0 µL of extracted DNA. Thermal cycler conditions (Bio-Rad T100) consisted of an initial 5-min denaturation at 95 °C followed by 35 cycles at 95 °C for 1 min, 58 °C for 1 min, 72 °C for 1 min and a final extension step of 72 °C for 7 min.

### Circumsporozoite (cs) ELISA for the detection of *Plasmodium *parasites in *Anopheles* spp.

The proportion of captured mosquitoes with sporozoites (sporozoite rate) was determined by performing circumsporozoite (cs) ELISA. The heads and thorax from all morphologically identified mosquitoes were processed and assayed to detect antibodies against the circumsporozoite proteins of *P. falciparum* (Pf), *P. vivax* VK210 (Pv210) or *P. vivax* VK247 (Pv247) using the sandwich csELISA according to the protocol established by Wirtz et al., (1992) [36] and updated methods from the 2017 Malaria Resource Reagent Reference Center [37]. False positives on the csELISA assay are known to appear in *Anopheles* spp. with cattle and swine blood meals as a result of cross-reacting antigens [38]. To avoid potential false positives, homogenate was boiled at 100 °C for 10 min to denature any heat-unstable cross-reactive proteins and retested to confirm positives.

## Results

**Fig. 1:**
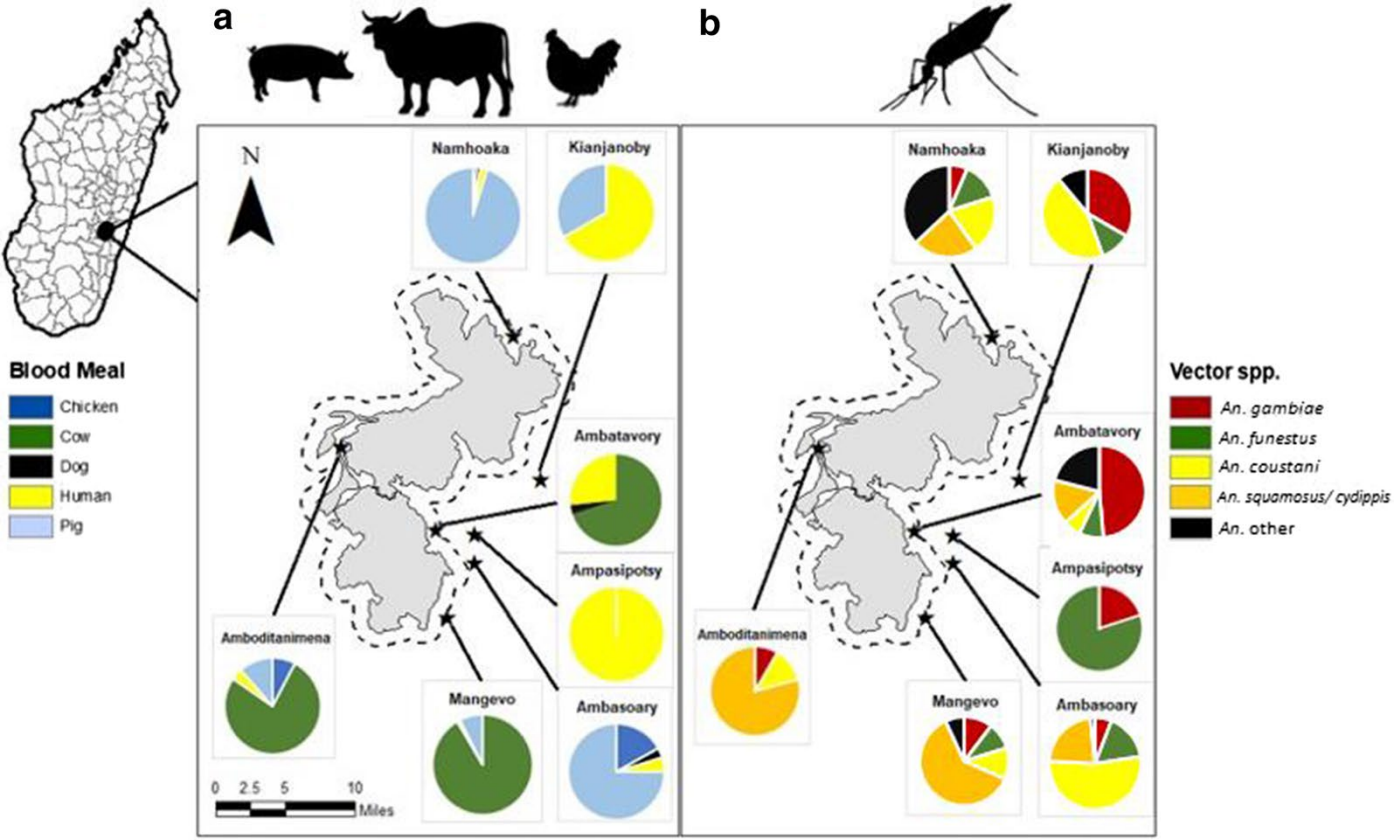
Mosquitoes were successfully assayed for blood meal composition (n = 480) were collected within Ranomafana National Park (park boundary in gray) or within 1 mile of the park boundary (represented by a dashed line) in the Ifanadiana District of Madagascar. **a** Blood meal source. Blood meals with multiple hosts identified are included in this figure as separate data points. **b** Vector species distribution

A total of 7762 *Anopheles* were collected. Of these, 7.5% (n = 582) had abdomens that contained a blood meal, as determined by visual inspection with a stereomicroscope. An equal proportion of blood fed mosquitoes were identified from specimens collected in both rainy and dry seasons. The blood fed mosquitoes were morphologically identified as 82 *An. gambiae s.l.* (14.1%), 71 *An. funestus s.l.* (12.2%)*,* 127 *An. coustani* (21.8%)*,* 214 *An. squamosus/cydippis* (36.7%)*,* and 88 unidentified *Anopheles* spp. (15.1%). The distribution of vectors was heterogeneous across all six survey sites, with greater diversity collected in the northern most and southern most points of Ranomafana National Park, and with *An. funestus s.l.* present in only five of the seven sites, *An. coustani* and *An. sqamosus/cydippis* collected in six of the seven sites, and *An. gambiae s.l.* captured in all seven. Specimens within *An. gambiae* and *An. funestus* complexes assayed using [32], and [34] to determine specific species were inconclusive due to the absence of positive amplicons for the targeted species specific sequences (Fig. 1).

### Blood meal identification

**Fig. 2:**
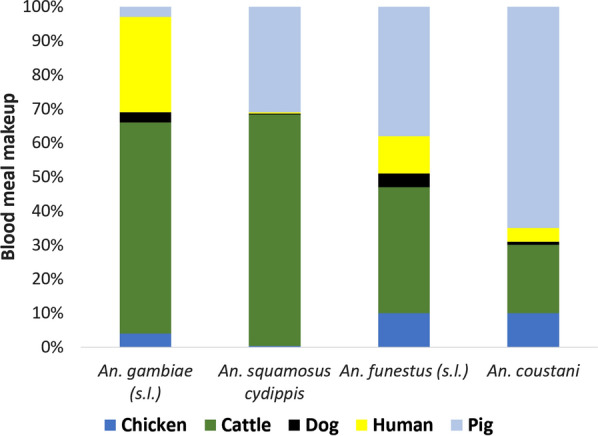
Blood meal analysis of *Anopheles* spp. in seven surveyed villages in southeastern Madagascar reveals high levels of zoophily in *An. gambiae s.l.* (n = 82), *An. funestus s.l.* (n = 71), *An. coustani* (n = 127) and *An. squamosus/ cydippis* (n = 214)

Our assays successfully identified bloodmeals from > 97% of all identified *Anopheles* spp. specimens assayed (n = 480). Among all *Anopheles* tested*,* a portion of all species tested displayed evidence of feeding on livestock hosts. Pigs and cattle were the source for most blood meals in villages close to the park, while humans accounted for a higher percentage of blood meals in villages further from the park boundaries. In total, > 92% of the blood meals taken by *Anopheles* collected in this study came from livestock and domestic animals, with cattle (245/480) and pigs (194/480) being the most common blood meal hosts. Human blood was detected in 7.5% (36/480) mosquito abdomens tested. Blood meals from cattle were detected in 71.8% of *An. squamosus/cydippis* (150/214), 56.4% of *An. gambiae s.l.* (44/78), 37.7% of *An. funestus s.l.* (26/69), and 20.2% of *An. coustani* (25/124; Fig. 2). Blood meals from pigs were second-most common among tested mosquitoes, with pig blood detected in 66.1% (82/124) of blood fed *An. coustani*, 43.4% of *An. funestus s.l.* (30/69), 33.5% of *An. squamosus/cydippis* (70/214), and 15.4% in *An. gambiae s.l*. (12/78). Finally, human blood was detected in < 10% of each *An. coustani* and *An. squamosus/cydippis*, but in 25.6% (20/78) and 11.6% (8/69) of *An. gambiae s.l.* and *An. funestus s.l.*, respectively (Fig. 2). Just over 5.4% (26/480) of all mosquito abdomens successfully assayed were positive for more than one type of blood meal, indicating multiple feedings (Table 1).

**Table 1:** Detection of multiple blood meals from different *Anopheles* spp. (n = 26) suggests potential plasticity of host choice in the Ifanadiana region of Madagascar

	Human	Cattle	Chicken	Dog	Pig	Cattle + Pig	Cattle + Human	Chicken + Human	Dog + Human	Pig + Human	Other
*An. gambiae s.l.*	19	44	2	1	11	0	0	0	1	0	1^a^
*An. funestus s.l.*	8	23	7	2	26	3	0	0	0	1	1^b^
*An. coustani*	5	25	9	2	78	0	0	1	0	1	3^a^
*An. squamosus*/ *cydippis*	0	136	1	0	58	12	1	0	0	0	1^c^

Other combinations: ^a^Chicken + Pig ^b^Dog + Pig ^c^Cattle + Dog

Of the 113 blood meals identified in mosquitoes trapped indoors, 8 (7.1%) were taken from human hosts while 105 (92.9%) were taken from livestock hosts. Human hosts accounted for 14 (31.1%) of the 45 blood meals identified in mosquitoes trapped near villages or rice fields and 14 (4.4%) of the 420 blood meals identified in mosquitoes trapped near livestock pens. Livestock hosts accounted for the remaining 31 (68.9%) blood meals identified in mosquitoes trapped near villages or rice fields, and the remaining 406 (95.6%) blood meals identified in mosquitoes trapped near livestock pens. Seven additional blood meals taken from livestock hosts were identified in mosquitoes trapped in forested sites or fallow fields (Table 2).

**Table 2:** Numbers and percentages of human vs. livestock blood meals at each trapping location type reveal many human blood meals identified in mosquitoes trapped outdoors and many livestock blood meals detected in mosquitoes trapped indoors

Collection location	# of human blood meals	% of human blood meals	# of livestock blood meals	% of livestock blood meals
Indoors (n = 113)	8	22.2	105	23.4
Village/ rice field (n = 45)	14	38.9	31	6.9
Livestock pen (n = 320)	14	38.9	306	68.1
Other sites (n = 7)	0	0	7	1.6
Total (n = 485)	36	100	449	100

### Plasmodium spp. infection

Only *An. gambiae s.l.* (n = 83) and *An. funestus s.l.* (n = 71) mosquitoes tested positive for *P. falciparum*, with a single infected specimen of each species detected. *Plasmodium vivax* 210 was detected in *An. gambiae s.l.*, *An. squamosus/cydippis* (n = 216) and *An. coustani* (n = 127) mosquitoes, with *An. coustani* demonstrating the highest sporozoite rate (> 3%). *Plasmodium vivax* 247 was found in *An. gambiae s.l*., *An. funestus s.l.*, *An. squamosus/cydippis* and *An. coustani* as well as a number of unidentified *Anopheles* spp. mosquitoes (n = 75). The sporozoite rate of *P. vivax* 247 among *Anopheles* spp. ranged from 4–16 mosquitoes. Coinfections between different *Plasmodium* strains were observed in a single *An. gambiae s.l.* (Table 3).

**Table 3:** Detection of *P. falciparum*, *P. vivax* 210 and *P. vivax* 247 in captured *Anopheles* spp. reveals high prevalence of infection with human malaria parasites, and mixed infections with *P. falciparum* and *P.vivax* 210 in *An. gambiae s.l.*

Species	*P. falciparum *+	*P. vivax* 210 +	*P. vivax *247 +	Mixed *Plasmodium *spp. infections
*An. gambiae s.l.* (n = 83)	1 (1.2%)	1 (1.2%)	6 (7.2%)	1 (1.2%)
*An. squamosus*/*cydippus *(n = 216)	0	1 (<1%)	16 (7.4%)	0
*An. funestus s.l.* ( n = 71)	1 (1.4%)	0	5 (7.0%)	0
*An. coustani *(n = 127)	0	4 (3.2%)	4 (3.2%)	0
*Anopheles *spp. (n = 75)	0	0	5 (6.6%)	0
Totals (n = 572)	2 (<1%)	6 (1%)	36 (6.3%)	1 (<1%)

## Discussion

### Vector species

This study found diverse communities of *Anopheles* species at study sites, including *An. gambiae* (*s.l.*), *An. funestus* (*s.l.*), as well as *An. squamosus/ cydippis* and *An. coustani*. The majority of visibly blood fed mosquitoes identified were species typically considered to be secondary vectors, particularly *An. coustani* and *An. squamosus/cydippis*. This is in line with other recent reports of *Anopheles* spp. in the Madagascar [39, 40]. *Anopheles coustani*, which comprised nearly 22% of blood fed mosquitoes, has been indicated as a new malaria vector of importance in Madagascar [41], while the role of *An. squamosus/cydippis* (over 37% of blood fed mosquitoes) in malaria transmission has long been suspected but less well understood [39, 41–44]. There were also relatively high numbers of common vector species identified, namely *An. gambiae s.l.* and *An. funestus s.l.*, which comprised over 14% and over 12% of blood fed mosquitoes, respectively. The species complexes were unable to be further analysed because of inconclusive PCR results. The only regional vector species of note not collected was *Anopheles mascarensis* [45]. Several specimens listed as unknown *Anopheles* spp. were morphologically similar to species not previously detected in Madagascar, and may have been incorrectly identified. Future taxonomic work on *Anopheles* mosquitoes in this region in Madagascar may shed more light on the region’s true morphological and molecular diversity.

### Blood meal diversity

These findings suggest overwhelmingly zoophagic behavior on the part of all *Anopheles* species collected, including the traditionally anthropophagic species *An. gambiae s.l.* and *An. funestus s.l.* The high level of livestock and animal blood feeding demonstrated by the *Anopheles* spp. captured outdoors here is in line with published descriptions of *Anopheles* host choice in Madagascar [39, 45]. As with other studies in the region, a high proportion of *An. gambiae s.l.* and *An. funestus s.l.* blood meals came from cattle (Fig. 2). In addition, almost as many *Anopheles,* most notably specimens of *An. funestus s.l.*, and *An. coustani* had fed on pigs (Fig. 2). Tedrow et al. also observed high numbers of pig blood meals among Malagasy *Anopheles* captured outdoors in December, 2017, although they observed a marked switch to human blood meals in April of the following year [39].

The detection of multiple blood meals, including mixed human-livestock blood meals (n = 5), indicates some degree of plasticity in host choice among both primary and traditionally secondar*y Anopheles* vectors in this region of Madagascar. This behavioural plasticity is further supported by mosquitoes with livestock blood meals indoors and mosquitoes with human blood meals near livestock pens. Previous studies of vector behaviour in the region suggest moderate to high levels of exophagy and exophily among many vector species [40, 41, 46], but these results are the first to indicate high levels of plasticity in feeding among these species. This plasticity may be in part due to heterogeneities in host availability or implementation of insecticide-based and other preventive measures in this region, as has been observed in other locations [18, 47]. This behavioural plasticity combined with high levels of zoophagy among all collected species points to a unique ecology among the *Anopheles* vectors of this region, one which requires new and integrative vector management strategies for effective malaria prevention.

Despite differences in trapping and molecular methods, the results presented here show predominant blood feeding on livestock by malaria vectors in Madagascar are consistent with Tedrow et al*.* [36]. While there were multiple blood meals detected within the same mosquito, using ELISA, there were far fewer mixed bloodmeals detected than observed by Tedrow et al*.* who used the BLOODART method- a PCR followed by multiplex bead assay, which may be more sensitive in detecting host DNA than direct ELISA [39]. In this study, odour-baited CDC light traps were used to target host-seeking mosquitoes. Trapping methods such as the QUEST method used by Tedrow et al*.* are passive and do not target host-seeking mosquitoes, but rather target outdoor resting mosquitoes which have already taken a blood meal [39], and therefore may better capture the extent to which multi-species feeding occurs among these vectors. The screening of all collected mosquitoes, i.e. inclusion of non-visibly blood fed mosquitoes, may yield more complete results. To monitor malaria control efforts, direct ELISA for human or bovine blood meal detection is commonly used to determine the human blood index (HBI) and bovine blood index (BBI) in a cost-effective way.

The majority of blood fed mosquitoes sampled, both those that had taken human and non-human blood meals, were collected from traps set outside of human habitations. In fact, over 50% of all blood fed mosquitoes collected in this study were trapped near livestock pens, away from human habitation. This suggests that Malagasy *Anopheles* mosquitoes may be particularly exophagic, a trend perhaps amplified by the successes of IRS and ITN strategies, which target endophilic mosquitoes. Host availability can influence feeding behaviour [18, 19], and these mosquitoes may be exhibiting plastic feeding behaviour, feeding largely on livestock, but switching to humans when available. Livestock and human densities may be important in interpreting blood meal composition in this context. These exophagic mosquitoes may be the key to understanding residual malaria transmission in Madagascar.

### Plasmodium spp. infection

*Plasmodium falciparum, P. vivax* 210 and *P. vivax* 247 (two different genotypes of *P.vivax*) were detected, with a higher than expected sporozoite rates of *P. vivax.* While the total prevalence of *P. falciparum* across all *Anopheles* specimens (0.35%; n = 572, n positive = 2) was fairly low, the higher prevalence of *P. vivax* 210 (1%; n = 572, n positive = 6) and *P. vivax* 247 (6.2%, n = 572, n positive = 36) may indicate that these parasites are being widely circulated in the region. These findings are contrary to current reports of malaria incidence in Madagascar, which state that *P. vivax* accounts for only 4% of the country’s human malaria burden [26]. One explanation for this may be that most *P. vivax* infections in Madagascar are either subclinical or unreported. *Plasmodium vivax* cases may also be commonly misdiagnosed as *P. falciparum* infection, due to the clinical similarities of infection and use of methods for diagnosis, such as rapid detection tests (RDTs) which only yield results of *P. falciparum*, non-*P. falciparum*, and mixed *Plasmodium* infection*.* Another potential explanation for the high prevalence detected in mosquitoes is that false positive sporozoite detection for *P. falciparum* and *P. vivax* by csELISA has been shown to be associated with bovine and swine blood meals [48]. Given the prevalence of bovine and swine blood meal in this dataset, it is possible that cross-reactive factors from the blood meals may influence our findings. To minimize the potential for false positives in this study, mosquitoes positive for *P. vivax* 247 were boiled, which has been shown to decrease cross-reactivity between blood proteins and *P. falciparum* [36], although its impact on *P. vivax* false positivity remains unresolved. Experimental laboratory studies using molecular strategies may elucidate this issue.

The status of *P. vivax* in Madagascar is unique. Over 80% of the population is thought to be Duffy-negative, meaning they express neither the Fy^a^ nor Fy^b^ antigens that allow infection by *P. vivax* [49]. However, in Madagascar, *P. vivax* clinical malaria is commonly observed among Duffy-negative individuals [50]. The high prevalence of *P. vivax* observed in *Anopheles* mosquitoes in this study further suggests the importance of considering *P. vivax* in malaria control programmes in southeastern Madagascar. In this study, *P. vivax* was detected in highly zoophilic and exophagic secondary vectors, such as *An. squamosus/cydippis*. While *An. squamosus/cydippis* has been suspected as a secondary vector of *P. falciparum* [42–44], this is the first documented evidence of their potential role in the transmission of *P. vivax*. Further investigation into the role of *An. squamosus/cydippis* and other secondary vectors in the transmission of *P. vivax* is needed.

## Limitations

These results provide evidence of zoophagy among *Anopheles* spp. malaria vectors in southeastern Madagascar; however, there are a few limitations to this work. For example, the collection method used here relied on CO_2_ as an attractant bait, while human-baited methods such as human landing catches (HLCs) or human baited light traps may have attracted more human biting vectors. Similarly, previous studies have found proximity to livestock pens to be a significant predictor of number of *Anopheles* spp. collected, therefore trapping near these sites may have influenced collection numbers. Additionally, PCR species identification results within the *An. gambiae* complex and *An. funestus* group were inconclusive, perhaps due to sample degradation. More accurate species identification may have revealed typically zoophagic species, such as *Anopheles arabiensis*. Further work characterizing species may provide additional insight into species specific trends in feeding behaviour. Due to cultural sensitivities and privacy around livestock ownership, accurate ratios of livestock to humans in these study sites, which may influence opportunistic feeding patterns, could not be determined.

### Zooprophylaxis and cattle in Madagascar

While there is still much debate about the potential of zooprophylaxis for malaria control [51–53], endectocide-aided zooprophylaxis, combined with IRS and ITNs, has proven to be effective in reducing residual malaria transmission by zoophilic vector species, especially in areas where these species make up a significant portion of the overall *Anopheles* spp. [51, 54–59]. Placement of livestock in pens away from housing and activity centers may draw behaviorally plastic feeders away from humans [57, 60]. Ingestion of endectocides during blood feeding from livestock can significantly reduce mosquito life-span and survival, thus reducing vector populations and lowering parity rates and transmission probability [55, 61–64]. This reduction in vector lifespan to a number of days below the extrinsic incubation period (EIP) of malaria disrupts the parasite’s transmission cycle, decreasing human cases of malaria [65]. This has mainly been studied in areas where *An. arabiensis* is the dominant vector species [56, 60, 66–68], but has the potential to be applied to any system with highly zoophagic, exophagic vectors [51, 54, 69, 70], providing a One Health solution to residual malaria transmission.

In Madagascar, despite overall reductions in malaria in recent decades [26], new approaches are necessary to combat the spread of malaria. The high level of zoophily described here and in other literature [39, 45] suggests potential exists to affect malaria transmission through some form of zooprophylaxis. The population of Madagascar primarily consists of rural, agrarian societies where cattle are significant [71]. The local cattle, known as *zebu*, are an extremely important part of Malagasy culture and society [71, 72], and may be used as insurance for periods of crop failures [73]. Furthermore, recent social and political instability has resulted in the rise of organized criminal gangs specialized in stealing zebu, leading to a change in traditional grazing regimes and movement of livestock from pastures to pens closer to human habitation [74]. Experimental studies have shown that the primary vector of malaria in the Central Highlands, *An. funestus*, preferred to seek out human odours to calf odors, while the primary vector of malaria at lower altitudes, *An. gambiae*, preferred calf odours [75]. Furthermore, another study indicated that traps near livestock pens were significantly more likely to capture *Anopheles* mosquitoes than those far from livestock pens [76]. This high level of zoophily among these and other Malagasy *Anopheles* species may grow in response to the ever-increasing population of livestock in Madagascar, as well as to recent changes in grazing regimes. The zoophily observed here combined with large numbers of cattle and swine in southeastern Madagascar living in close proximity to communities, may make this location an ideal intervention site for the application of zooprophylaxis using endectocides to treat livestock [27, 48, 76]. This would simultaneously function as a veterinary health preventive measure for helminth infections and lead to healthier livestock while controlling malaria vector populations.

## Conclusion

Understanding local vector species composition, host choice and sporozoite rates allows for improved malaria control efforts, tailored to the transmission dynamics of the locality. There were large numbers of *Anopheles* species typically considered to be secondary vectors of malaria identified, as well as evidence of *Plasmodium* spp. sporozoites among both primary and secondary vector species and high levels of zoophagy among all species of malaria vectors collected in our study area in southeastern Madagascar. These findings, combined with evidence of mixed human-livestock blood feeding in this region, suggest potential for the use of tools such as endectocide-aided zooprophylaxis, to complement current prevention efforts. Also, a high sporozoite rate of *P. vivax* was found in assayed *Anopheles* spp., which contradicts the low reported cases of *P. vivax* in humans in Madagascar. This discrepancy between observed sporozoite rates and reported human cases may in part be due to high Duffy negativity in Malagasy populations and a high number of asymptomatic individuals not seeking treatment and therefore continuously contributing *P. vivax* into the ecosystem. Future vector surveillance in Madagascar is suggested to include surveillance of *P. vivax*. Finally, malaria prevention efforts in southeastern Madagascar are suggested to explore the use of tools such as endectocide-aided zooprophylaxis to supplement other efforts in this region.

## Data Availability

All data generated are included in this manuscript and supplementary files.

## Abbreviations

BLOODART: Bloodmeal detection assay for regional transmission
CS: Circumsporozoite
DNA: Deoxyribonucelic acid
ELISA: Enzyme-linked immunosorbent assay
ITN: Insecticide treated bed nets
IRS: Indoor residual spraying
PCR: Polymerase chain reaction
QUEST: Quadrant enabled screen trap
